# Assessing Intellectual Disability in Culturally and Linguistically Diverse Adults: A United Kingdom Delphi Study

**DOI:** 10.1111/jar.70279

**Published:** 2026-07-09

**Authors:** Eimear McCabe, Freddie O'Donald, Doug McConachie, Clara Calia

**Affiliations:** ^1^ Child and Adolescent Mental Health Services, NHS Grampian Aberdeen UK; ^2^ School of Health in Social Science, University of Edinburgh Edinburgh UK; ^3^ Centre for Child Health, NHS Tayside Dundee UK; ^4^ NHS Education for Scotland Edinburgh UK

**Keywords:** cross‐cultural assessment, cultural and linguistic diversity, Delphi methodology, diagnostic equity, interpreter‐mediated assessment

## Abstract

**Background:**

United Kingdom (UK) clinicians report growing demand for intellectual disability diagnostic assessment among adults from culturally and linguistically diverse backgrounds, yet practice is variable, and evidence to guide assessment is limited. This study aimed to establish expert consensus on research, policy, and clinical practice priorities.

**Method:**

A mixed‐methods Delphi design was used, comprising three rounds. Twenty UK‐based psychology experts met the inclusion criteria and completed iterative online surveys. Round 1 responses were analysed using template‐based thematic analysis to generate statements for rating in subsequent Delphi rounds.

**Results:**

Of 32 statements, 16 (50%) reached consensus. Priorities included developing and validating cross‐culturally applicable cognitive tools, evaluating interpreter‐mediated assessment, strengthening the measurement of cross‐cultural adaptive behaviour, and improving service conditions and training.

**Conclusions:**

Improving equity in intellectual disability assessment requires methodological and service‐level resourcing and guidance to support best practice.

## Abstract (plain language summary)

**Lay Summary**

- Psychologists in the UK agree that assessing intellectual disability in adults from diverse cultural and linguistic backgrounds is not well supported by existing tools or guidance.
- Many commonly used tests were developed for English‐speaking Western populations and may yield unfair or misleading results when used with people from other cultural or linguistic backgrounds.
- Experts agreed that fairer assessments require better tools, clearer guidance, trained interpreters, and more time and support from services, not merely better individual clinician skills.
- For research, the findings highlight urgent priorities for developing and testing more culturally appropriate assessment methods so that people with intellectual disabilities can access support and services more equitably.

## 1 Introduction

Adults from culturally and linguistically diverse backgrounds in the United Kingdom (UK) face well‐documented inequalities in access to healthcare, quality of assessment, and health outcomes (Kapadia et al. 2022). When intellectual disability is present or suspected, these inequalities are compounded, as diagnosis functions as a gateway to specialist services, legal protections, and long‐term support (Sonderlund et al. 2024). Barriers to accurate and timely diagnosis, therefore, have far‐reaching consequences, shaping not only clinical care but also broader social inclusion, autonomy, and life opportunities.

These challenges arise in a rapidly diversifying demographic context. Over recent decades, the UK has become increasingly ethnically, culturally, and linguistically heterogeneous, with census data showing steady growth in populations identifying with minority ethnic backgrounds, alongside rising multilingualism and cultural heterogeneity (Office for National Statistics 2021). These demographic shifts have direct implications for health and social care services. By 2030, approximately 25% of those newly entering adult social care for intellectual disability (ID) are expected to be from minority ethnic backgrounds (Emerson 2012), while referrals for adult intellectual disability assessments from minoritised communities continue to increase (Whitaker 2018). Together, these trends expose a mismatch between service demand and assessment models largely developed for more culturally homogeneous populations.

Culture and ethnicity are often used interchangeably in research, despite being distinct yet overlapping constructs (Durà‐Vilà and Hodes 2009). Culture refers to shared patterns of values, beliefs, behaviours, and meanings learned and transmitted within social groups (Birukou et al. 2013), whereas ethnicity is a multifaceted construct shaped by historical, social, and political processes and may be self‐identified or externally ascribed (Liebkind 2006). Considerable variation can exist within ethnic groups, and acculturation varies across individuals and contexts (O'Donald and Calia 2025a). Both culture and ethnicity are associated with socioeconomic disadvantage and inequalities in healthcare access (Stancliffe and Lakin 2006) and influence help‐seeking behaviour, beliefs about disability, stigma, and engagement with services (Allison and Strydom 2009; Durà‐Vilà and Hodes 2009). Consequently, individuals from minoritised ethnic backgrounds often encounter barriers to healthcare that extend beyond language differences alone.

Estimates of the prevalence of intellectual disability across cultures vary widely, typically ranging from 2% to 8%, though the ‘true’ prevalence is difficult to establish because of variation in diagnostic criteria, social contexts, and assessment practices (Leonard and Wen 2002; Maulik et al. 2011). UK‐based studies suggest that overall prevalence among minority ethnic groups is broadly comparable to that of White British populations (McGrother et al. 2002), although some evidence points to higher rates among South Asian communities (Emerson et al. 1997; Robertson et al. 2019). Irrespective of prevalence, individuals from minoritised backgrounds with intellectual disability experience compounded vulnerability, arising from intersecting forms of discrimination related to disability and ethnicity (Allison and Strydom 2009), with poorer access to services and poorer health outcomes consistently reported (Kapadia and Bradby 2021). Central to these inequities is the diagnostic process itself: without a valid and trusted diagnosis, access to appropriate support is severely constrained (NHS Race and Health Observatory 2023).

In the UK, intellectual disability is often identified during childhood through paediatric services, although it may also be recognised for the first time in adulthood. Diagnosis is based on DSM‐5 or ICD‐11 criteria and relies on standardised cognitive assessment, most commonly in adults, the Wechsler Adult Intelligence Scale–Fourth Edition (WAIS‐IV), alongside measures of adaptive behaviour, developmental history, and clinical judgement (BPS 2015).

Despite the developmental criterion, intellectual disability may, in some cases, be identified for the first time in adulthood, particularly where earlier access to assessment has been limited or where individuals have migrated to the UK after the developmental period, experienced limited access to education or assessment, or presented with borderline or mild difficulties that did not meet thresholds or were attributed to language, educational, or cultural factors (Emerson 2012; Whitaker 2018), thereby making assessment a core function of adult intellectual disability services.

Although guidance increasingly recognises the limitations of rigid intelligence quotient (IQ) thresholds (Ardila 2005; Whitaker 2008), practice continues to rely heavily on measures normed predominantly in Western, educated, English‐speaking neurotypical populations (Shuttleworth‐Edwards 2016; O'Donald et al. 2025). This poses particular challenges when assessing individuals from culturally and linguistically diverse backgrounds. Limited proficiency in the language of assessment can confound test performance, complicating distinctions between cognitive impairment and language‐related barriers (Mindt et al. 2019), a risk further amplified in bilingual and multilingual populations (Franzen et al. 2022). While interpreter use is recommended (BPS 2017), interpreter‐mediated cognitive assessment introduces ethical and methodological challenges, including altered stimulus equivalence, reduced behavioural observation, and variability in interpreter expertise (Fujii et al. 2022; O'Donald and Calia 2025b). Even when language barriers are addressed, cultural bias may remain embedded in many cognitive measures, with educational access, test familiarity, and culturally specific knowledge influencing performance on both verbal and non‐verbal tasks (Calia, Nielsen, et al. 2026; Metcalfe et al. 2026; Nielsen and Staios 2023). Although the WAIS‐IV is widely used in intellectual disability assessment due to its standardisation and psychometric robustness, no adult cognitive measure currently offers global normative applicability or cultural invariance.

An accurate diagnosis also requires evidence of significant limitations in adaptive behaviour, defined as the skills needed to meet the demands of everyday life within a given social and cultural context (Schalock 2004). Despite international progress in adapting adaptive behaviour measures, tools used in the UK remain largely normed on Western populations (AlMuhairy et al. 2023; Oakland et al. 2013). Cultural norms regarding independence, gender roles, and family responsibility vary widely, raising concerns about validity when these measures are applied across cultures (Calia et al. 2025). Questionnaire‐based informant reports may be particularly vulnerable to cultural bias and misinterpretation, especially when administered through interpreters (Durà‐Vilà and Hodes 2009).

Although professional guidance recognises the need for cultural sensitivity and clinical judgement when assessing intellectual disability in culturally and linguistically diverse populations (BPS 2015), recommendations remain broad and open to interpretation, leading to variability in practice across clinicians and services. Comparable challenges have been addressed more systematically in other areas of neuropsychology, particularly dementia, where structured expert consensus has informed research priorities and clinical guidance (Franzen et al. 2022). In contrast, there remains a notable absence of equivalent work on intellectual disability, despite the unique ethical, developmental, and service‐access implications of diagnosis. While existing guidance outlines broad principles, it provides limited detail on how to apply these in complex, real‐world assessment settings. In practice, clinicians often need to make diagnostic decisions when standardised procedures cannot be reliably implemented, yet there is little agreement on what constitutes defensible practice in such situations.

Many of the challenges described are also relevant in child and adolescent services, where cross‐cultural neuropsychological assessment has received increasing attention (O'Donald et al. 2025). However, assessment in adulthood introduces additional complexities. These include the need to retrospectively determine developmental onset, often in the absence of prior formal assessment, often with limited access to reliable informants or documentation, and to interpret functioning within the context of diverse educational and migration histories. In adult services, diagnosis also plays a key role in determining eligibility for support, services, and legal protections. Despite this, there remains relatively limited research specifically addressing the assessment of intellectual disability in culturally and linguistically diverse adults, which underpins the focus of the present study.

Given the complexity of these assessments, the absence of standardised solutions, and reliance on expert judgement, there is a clear need to establish consensus among experienced clinicians on priorities for research, policy development, and clinical practice. The present study, therefore, employed a Delphi methodology to systematically explore expert opinion on the assessment of intellectual disability in adults from culturally and linguistically diverse backgrounds in the UK.

## 2 Methods

### 2.1 Study Design and Delphi Technique

A mixed‐methods Delphi approach was used to explore expert opinion and establish consensus on the assessment of intellectual disability in adults from culturally and linguistically diverse backgrounds. In the present study, the Delphi process was combined with qualitative thematic analysis to generate content grounded in clinical experience and to subsequently quantify expert consensus (Roberts et al. 2019).

### 2.2 Participants and Definition of Expertise

A core element of the Delphi method is the selection of an appropriate expert panel. Following guidance from Baker et al. (2006), expertise in this study was defined in relation to the research topic rather than formal subspecialist accreditation. Participants were eligible for inclusion if they met all of the following criteria:

- Were a Health and Care Professions Council (HCPC), Association of Clinical Psychologists (ACP), or British Psychological Society (BPS) registered psychologist working in the UK, or an academic psychologist specialising in intellectual disability assessment and cross‐cultural research.
- Currently working, or having worked within the past 5 years, in a role involving the assessment of intellectual disability in adults.
- Had completed at least one assessment for intellectual disability within the past 5 years, involving an adult whose first language was not English or who was from a culturally and linguistically diverse background.

There is limited evidence that larger panel sizes improve the reliability or validity of Delphi findings; instead, panel quality and the relevance of expertise are often considered more important (Rowe and Wright 1999). Previous studies suggest that panels of 10–15 experts are sufficient to generate robust consensus, although sample sizes vary widely in practice (Nasa et al. 2021). Given that intellectual disability assessment in the UK is primarily conducted by practitioner psychologists operating under broadly similar professional standards (Förster and von der Gracht 2014), the expert group was expected to be relatively homogeneous in core competencies. However, some diversity in experience and service context was sought, as this is considered beneficial in Delphi studies (Beiderbeck et al. 2021). To account for potential attrition and in line with recommendations that up to 30% attrition can be acceptable (Keeney et al. 2010), we aimed to recruit 20 experts, with a minimum of 14 completing all rounds.

### 2.3 Recruitment and Sampling Strategy

Recruitment was conducted through three primary routes. First, the study was advertised on professional social media platforms, including Facebook, LinkedIn, Instagram, and X. Second, the first author contacted team leads in intellectual disability services across the UK by email, providing study information and offering to attend team meetings to introduce the project. Third, participants were encouraged to share study details with eligible colleagues in their professional networks. Members of the wider research team also disseminated the study within their networks.

### 2.4 Procedures

All data were collected via online surveys hosted on the JISC platform. Participants accessed the study through a link in the recruitment materials, which directed them to the Participant Information Sheet and consent form. Eligibility for participation was verified at enrolment. The study comprised three Delphi rounds.

Round 1, the exploratory qualitative phase, comprised demographic questions and open‐ended qualitative items. Demographic information included years of experience, the service or geographical area of work, and the ethnic, cultural, or linguistic backgrounds of client groups commonly assessed. Open‐text responses were used to capture client background information, avoiding the imposition of rigid or hierarchical ethnic categories (Lam et al. 2023).

The qualitative component invited participants to reflect on barriers encountered when assessing adults from culturally and linguistically diverse backgrounds for intellectual disability, including challenges in cognitive assessment, adaptive functioning, developmental history, and the use of interpreters. Participants were also asked about current practices, perceived gaps in knowledge or resources, and priorities for future research and training. These questions were informed by existing literature and policy guidance and were designed to allow broad exploration and idea generation.

Responses from Round 1 were analysed using template‐based thematic analysis (King 2012). A preliminary coding template was developed from relevant policy guidance, the existing literature, and an initial familiarisation with the data. The template comprised four overarching themes and 20 initial codes, which were applied deductively to the data. Subsequently, inductive coding was undertaken to capture concepts not accounted for in the initial template, resulting in the development of an additional 24 codes. Initial coding was conducted manually in NVivo 12. Across all data, 268 coded segments were identified under 44 codes, which were iteratively refined into four final themes and ten subthemes. The final codebook was applied across the dataset to assess saturation and consistency. The themes derived from this analysis were used to generate statements for the subsequent Delphi rounds.

In Round 2, participants rated the statement generated. Thirty‐two statements derived from the Round 1 thematic analysis were presented in Round 2. Participants were emailed a survey link and given 3 weeks to respond.

Participants were asked to rate 22 statements on a five‐point Likert scale from “1 = Not at all important” to “5 = Extremely important,” and a further ten statements on a five‐point scale from “1 = Strongly disagree” to “5 = Strongly agree.” Free‐text comment boxes were provided to allow participants to elaborate on or qualify their responses. Statements that reached marginal consensus (defined as 70%–85% agreement) were retained for re‐evaluation in the next round.

Round 3, consensus confirmation, focused on the seven statements that reached marginal consensus in Round 2. Participants were given 5 weeks to complete this round.

For each statement, participants received anonymised group feedback, including the percentage of responses at each Likert point, the median, mean, and standard deviation, their previous response, and any qualitative comments from other participants. Participants were then asked to re‐rate each statement. No new statements were introduced at this stage.

The number of Delphi rounds was determined a priori. In line with previous Delphi studies in healthcare research, two to three rounds are usually sufficient to achieve response stability while reducing participant attrition. A three‐round design was chosen to allow initial idea generation, quantitative rating, and re‐evaluation of items as they approached consensus.

### 2.5 Consensus Criteria

There is no universally accepted standard for defining consensus in Delphi studies, and thresholds vary by study aims and sample size. Given the relatively small panel, a stringent threshold (≥ 85%) was adopted to ensure robustness of agreement and sensitivity to divergent views, consistent with recommendations for Delphi studies in healthcare research (Earley 2015). Consensus criteria were defined a priori as follows:

1. Statements were considered to have reached consensus if ≥ 85% of participants rated the item as “agree” or “strongly agree,” or as “important” or “very important.”
2. Statements receiving 70%–85% agreement were carried forward to Round 3 for re‐evaluation.
3. Statements that did not meet either criterion were considered to reflect divergence of opinion

Items that did not reach consensus after Round 3 were retained and reported as areas of divergence, reflecting ongoing uncertainty or variability in clinical practice, rather than being excluded from analysis.

## 3 Results

Three sets of results are presented, with key findings indicating limited use of culturally appropriate tools, variability in assessment practice, and strong consensus on the need for improved guidance and resourcing. First, descriptive data summarise participant demographics and current clinical practice. Second, qualitative findings from the template‐based thematic analysis are presented, integrating data across Delphi rounds. Third, quantitative results from the Delphi process identify statements that achieved expert consensus.

### 3.1 Participant Characteristics

#### 3.1.1 Demographics of Experts

Twenty‐two participants completed the initial Round 1 qualitative questionnaire. Two participants were excluded from further analysis because they did not meet the inclusion criterion of prior experience in assessing intellectual disability in individuals from culturally and linguistically diverse backgrounds. The final sample, therefore, comprised 20 experts.

Participants were based across the UK, including NHS Scotland (*n* = 14), NHS England (*n* = 5), and NHS Wales (*n* = 1). Nineteen participants were qualified clinical psychologists, and one was a Clinical Associate in Applied Psychology. The majority of participants were highly experienced clinicians, with 75% reporting more than 10 years' experience in intellectual disability services. A further 10% reported 3–5 years' experience, 10% reported 1–2 years' experience, and 5% reported less than 1 year's experience. No participants reported 6–10 years' experience.

The mean number of assessments conducted per year with adults from culturally and linguistically diverse backgrounds was 3.65 (SD = 3.32), with responses ranging from fewer than one assessment per year to monthly assessments. Qualitative responses were converted into annualised estimates for consistency (e.g., “once per month” was recorded as 12 assessments per year).

#### 3.1.2 Ethnic, Cultural, and Linguistic Groups Seen in Practice

Participants were asked to report the most common ethnic, cultural, or linguistic backgrounds of clients they assessed, excluding british or irish backgrounds. Free‐text responses were used instead of predefined categories to avoid imposing rigid ethnic classifications and to better reflect the complexity of cultural identity. As participants commonly worked with individuals from multiple backgrounds, frequencies reflect the number of mentions rather than the number of participants.

Clients from asian backgrounds were most frequently reported, with 22 mentions across participants. Within this group, Indian (*n* = 8) and Pakistani (*n* = 7) backgrounds were most commonly cited. Urdu (*n* = 6) and Bengali (*n* = 4) were the most frequently mentioned languages, with references to Bangladeshi (*n* = 3) and Kashmiri (*n* = 2) Regions. Arabic was also commonly reported (*n* = 5), with references to several Arabic‐Speaking countries. European backgrounds were the second most frequently reported group, with 16 mentions across participants. Within this category, Polish Nationality was most commonly cited (*n* = 11). There were four mentions of clients from african backgrounds and one of clients from the Caribbean. In addition, two participants reported conducting assessments through British sign language, underscoring its recognition as a distinct minoritised linguistic and cultural group.

#### 3.1.3 Use of Interpreters

**FIGURE 1:**
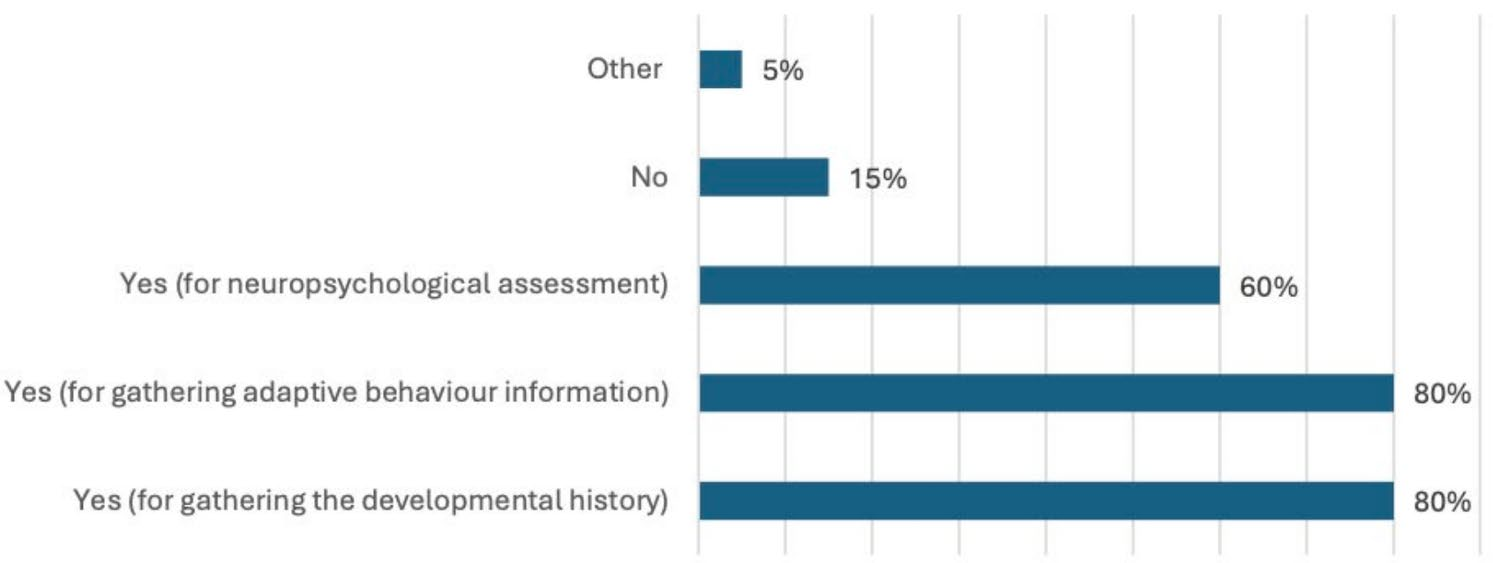
Participant experiences of using interpreters in the assessment process.

Seventeen participants reported experience of using professional interpreters at some stage of the intellectual disability assessment process. Interpreter use was most common for gathering developmental history and adaptive functioning information, and less frequent during formal cognitive assessment. Patterns of interpreter use are illustrated in Figure 1.

#### 3.1.4 Use of Alternative Assessment Measures

Fewer than half of participants (*n* = 9) reported using alternative or culturally adapted assessment measures instead of the WAIS‐IV when assessing for an intellectual disability in culturally and linguistically diverse populations. Reported alternatives included the Leiter‐3 (*n* = 2), Raven's progressive matrices (*n* = 1), the test of non‐verbal intelligence (TONI; *n* = 1), the wechsler non‐verbal scale of ability (*n* = 1), and non‐verbal scales used alongside occupational therapy or speech and language therapy input (*n* = 2). The majority of participants (*n* = 11) reported either no access to or not using alternative assessment measures.

Overall, these descriptive findings suggest that assessments are often carried out with limited access to culturally adapted tools and inconsistent use of interpreters, emphasising reliance on standard methods that may not Be suitable for culturally and linguistically diverse populations.

### 3.2 Thematic Analysis Results

Thematic analysis identified four overarching themes that reflect key areas of need and uncertainty in current practice: (1) Priorities for Research, (2) Policies and Guidelines, (3) Clinical Practice, and (4) The Clinical Context. Themes, subthemes, and definitions are summarised in Table 1. Subthemes are described below and illustrated with extracts from participant responses. Across these themes, three key findings emerged: that current assessment tools are perceived as culturally limited, that guidance is inadequate to support consistent practice, and that additional time and structural support are necessary to perform valid assessments.

**TABLE 1:** Evolutions of themes and their descriptions.

Initial theme	Sub themes	Definitions
Priorities for research	Culture‐free cognitive assessments	A theme was generated about the need for further research in this area, identifying priorities such as validating additional assessment tools, markers for differential diagnosis between trauma and intellectual disability presentations, research into cultural differences in intellectual disabilities as a construct, and the conceptualisation of adaptive behaviour. The theme highlights the bias towards validating measures in Western‐educated populations, as well as the limited understanding of intellectual disabilities in other cultures. Additionally, research into the experiences of this population being assessed for intellectual disability within the United Kingdom. The theme demonstrates the barriers faced in assessment due to limited research in these areas. The iteration encompasses the expert's acknowledgment of limitations and problems within the current evidence base.
	Adaptive behaviour and culture	
	The client's experience	
Policies and guidelines	Updating policy	At a macro level, existing policies and guidelines for assessing intellectual disability in culturally and linguistically diverse populations appear vague and open to interpretation by clinicians. A theme emerged that clinicians felt they needed to work in a non‐standardised way with this population, yet policy does not provide clear guidance on how to do so. In addition, there is a lack of literature on the use of multidisciplinary approaches with this population. This also highlights the need for policy and guidelines to be updated to reflect existing knowledge and ensure consistency across services.
	Changing policy	
Clinical practice	Clinicians overcoming barriers	Within this theme, clinicians have shown how they make novel use of the resources they have within themselves and the system to assess the population. Clinicians outline examples of utilising clinical judgement, using standardised assessments in non‐standardised ways, thinking outside the box and drawing on expertise from other sources. This theme is understood as the individual effort clinicians employ to overcome the barriers of the assessment process.
	Skills and training	
Clinical context	Time	A theme was identified that highlighted structures within the clinical practice system as barriers to completing assessments for people with intellectual disabilities. This included practical elements, such as time pressures, lack of access to appropriate tools, and appropriate training for staff members. Within this theme, additional barriers related to interpreters were also identified, including reliability, time to brief interpreters, and consistency.
	Diversity in employment	
	Multidisciplinary input	

#### 3.2.1 Theme 1: Priorities for Research

This theme highlights three main priorities: (i) reducing cultural bias in cognitive assessment, (ii) improving the cultural validity of adaptive behaviour assessment, and (iii) increasing understanding of how assessment is experienced across cultures. Overall, this theme appears to reflect participants' shared perception that the current evidence base offers limited support for assessing intellectual disability in adults from culturally and linguistically diverse backgrounds. Three subthemes were identified: culture‐free cognitive assessments, adaptive behaviour and culture, and the client's experience. Taken together, these findings indicate that current approaches to both cognitive and adaptive assessment are not perceived as culturally valid.

##### 3.2.1.1 Culture‐Free Cognitive Assessments

Current cognitive assessments were consistently recognised as culturally embedded and limited in their relevance across different populations, emphasising that most are validated on Western, mainly White populations and contain assumptions about education, speed, and test familiarity. Several participants expressed concern that such measures might underestimate ability among individuals educated in different systems.

Participants consistently highlighted concerns that existing assessments are shaped by Western educational assumptions. For example: “Neuropsych assessments measure ability based on assumptions made in the European/American school systems, e.g., that speed and accuracy are of utmost importance, but that these assumptions may not be true of education in other areas of the globe, and that as a result neuropysch tests can under‐estimate the ability of people who grew up in other places” (P04).

While some participants reported using assessments perceived as more culturally neutral (e.g., the Leiter), these were not considered truly culture‐free. Suggestions included validating existing measures across cultures or developing authorised substitutes within those tests.

Participants also identified the need for culturally appropriate adaptations within existing measures to reduce reliance on culturally specific knowledge. For example: “It would be useful to be able to have some culturally appropriate alternatives to certain questions, for example for the question about Hamlet author on information, a well known book taught in schools of different countries could be compiled to enable some robust and authorised substitutions based on curriculums used in other countries” (P17).

Overall, participants emphasised the need for assessments that measure underlying cognitive constructs without relying on culturally learned knowledge. More broadly, participants emphasised the importance of developing approaches that minimise culturally embedded knowledge demands. For example: “I don't think the issue is the requirement of culture specific normative data, so much as moving away from tests that are culturally embedded, towards culture‐free testing, i.e., tests that are applicable cross‐culturally (without bias towards any one specific culture, population, language, etc.)” (P15).

While framed by participants as a need for “culture‐free” approaches, this could also be seen as a concern about reducing culturally mediated variance in test performance, rather than expecting assessment to be entirely independent of cultural context.

##### 3.2.1.2 Adaptive Behaviour and Culture

Participants identified significant gaps in understanding how adaptive behaviour varies across cultures and expressed concerns about applying existing measures without cultural adaptation. They frequently cited differences in gender roles, family expectations, and opportunities to develop specific skills.

Participants described how culturally patterned roles and expectations can influence adaptive behaviour scores. For example: “In certain cultures, adults still living in their family home, especially men, would not be expected to know how to do things around the house (such as finding a plumber, or knowing how to use the washing machine), so lower scores on the test would not necessarily mean that they lack the ability rather they have never had the chance to develop the skills.” (P04).

Rather than calling for culture‐free measures of adaptive behaviour, participants emphasised the need for broader assessment approaches, including observation and contextualised clinical judgement, looking at the individual across the lifespan.

Participants emphasised the importance of contextual and observational approaches when interpreting adaptive functioning. For example: “In my experience, adaptive behaviour assessments that solely rely on questionnaires are not reliable. In my view these should be based on observations. There should also be information about upbringing, opportunities to learn certain skills, family circumstances, roles etc. that would let the assessor make a judgement ln whether the individual actually had the opportunity/encouragement to develop certain adaptive behaviour skills.” (P09).

These accounts imply that adaptive functioning must be understood in relation to culturally specific expectations, prompting questions about whether current constructs truly measure ability or merely opportunity.

##### 3.2.1.3 The Client's Experience

Participants highlighted limited understanding of how individuals and families from culturally and linguistically diverse backgrounds experience the assessment process. Intellectual disability was recognised as a culturally situated construct, with interpretations varying across societies.

Participants highlighted variation in how intellectual disability is understood across cultures. For example: “How autism and LD [intellectual disability] are considered in other cultures and countries, understanding of support vs ‘cures’” (P11).

Participants also described risks arising from inaccurate assumptions due to limited cultural knowledge. For example: “Less knowledge of individual's culture, education system, healthcare system–increases risk of forming inaccurate assumptions” (P18).

Some participants suggested that the assessment process may be confusing or unfamiliar to families and reflected on how the assessment process may be experienced as confusing or unfamiliar. For example: “I suspect people being assessed and family members find the whole process very confusing” (P06).

Taken together, these findings suggest that assessment is not only a technical process but also a culturally mediated interaction, in which meaning, expectations, and understanding can differ significantly between clinicians and families.

#### 3.2.2 Theme 2: Policies and Guidelines

This theme captured concerns about the adequacy of current policy and professional guidance. Two subthemes were identified: Updating policy and changing policy.

##### 3.2.2.1 Updating Policy

Participants described the existing guidance as vague and open to interpretation, resulting in inconsistent service provision. They described limited clarity in existing guidance, for example: “In general, clarifications whether we can/should assess individuals where we can't use standard procedure, as lack of guidance is leading to inequality in service provision (some services/Psychologists agree to assess individuals from other ethnical/language backgrounds, and other don't)” (P09).

Participants reported uncertainty about how to operationalise “clinical judgement” when standardised assessment is not feasible and expressed a need for clearer guidance on integrating qualitative information into diagnostic decision‐making. For example: “The BPS guidance recommends the use of clinical judgement in situations where we cannot use the WAIS‐ it would be helpful for there to be more research that picks apart what this means, what factors are most important etc.” (P03).

##### 3.2.2.2 Changing Policy

Some participants questioned reliance on fixed IQ thresholds and proposed a more formulation‐based approach to intellectual disability. Others raised concerns about feasibility, service capacity, and clarity for families and non‐psychological professionals.

Participants often questioned the reliance on fixed diagnostic thresholds and suggested alternative approaches. For example: “My first thought was developing more tools for assessing people from that population. My second thought was that IQ testing is a social construct and creating a model where we attribute to people LD and non‐LD label can be quite counterproductive, and at times stigmatizing. I would hope that eventually we could move from a service model that relies on diagnoses to one that actually uses formulation as a way of assisting individuals.” (P04).

Overall, findings across Theme 2 highlight uncertainty and inconsistency in how existing guidance is interpreted and applied, suggesting that current frameworks may be inadequately specified to support equitable practice across diverse contexts.

#### 3.2.3 Theme 3: Clinical Practice

This theme reflects how clinicians adapt practice to overcome assessment barriers. Subthemes included clinicians overcoming barriers, skills, and training.

##### 3.2.3.1 Clinicians Overcoming Barriers

Participants described adapting standardised assessments, combining multiple sources of information, and relying on qualitative observation. For example: “I use a lot more observational and qualitative information, e.g., what is there approach to solve a problem? Can they adapt? Can they transfer one solution to another one? Can they remember what we did at previous sessions? What are there difficulties in every day life? How did they manage to adapt to living in the UK?” (P09).

Adaptations were described as requiring a strong understanding of what tests measure and how results can be interpreted flexibly. Others described combining multiple assessment methods to support interpretation. For example: “When I have used a WAIS I have switched the information subtest for comprehension I have used the TONI (Test of Non‐Verbal Intelligence) to place less emphasis on a person's understanding of language. When I have interpreted the scores, I have then looked at both the WAIS and TONI results, plus usually an adaptive functioning scale, and drawn a tentative conclusion.” (P07).

##### 3.2.3.2 Skills and Training

Participants held differing views on whether trainees and assistant psychologists should conduct these assessments, with most emphasising the importance of supervision. Key skills identified included cultural sensitivity, flexibility, curiosity, and confidence in formulation. Participants also highlighted the need for training in working with interpreters and for interpreters to receive training relevant to neuropsychological assessment.

Participants identified key clinician skills required when working in this context. For example: “Patience and time to get to know the person. Helpful to get information from different sources. Awareness of the impact of trauma and discrimination on functioning. Ability and confidence to rely on clinical formulation, rather than psychometric assessment in coming to decision about eligibility for specialist learning disability services” (P05).

These findings from Theme 3 indicate that clinicians depend heavily on adaptation, tacit knowledge, and individual judgement, reflecting a move away from standardised assessment towards more interpretive practices in the absence of adequate tools and guidance.

#### 3.2.4 Theme 4: The Clinical Context

This theme captured the systemic and structural factors influencing assessment. This included access to resources and availability of time or additional professional input. This theme includes the subthemes time, multidisciplinary input, and diversity in employment.

##### 3.2.4.1 Time

Participants consistently reported that assessments with this population require additional time, including interpreter briefing, longer appointments, and extended information gathering. For example: “Allowing more time at each stage of process e.g., initial session with interpreter prior to session with patient and family, extended interview with patient and family, allowing time for interpretation” (P13).

##### 3.2.4.2 Diversity in Employment

Participants highlighted the value of workforce diversity and shared cultural knowledge, while acknowledging recruitment challenges and proposing regional advisory support models.

Participants also highlighted structural challenges related to workforce diversity and access to expertise. For example: “It's a very hard to recruit to area so whilst we can aspire for diversity very often we are limited to the one applicant for the job. It would be helpful if there was a pool of people that you could seek advice/support from” (P08).

##### 3.2.4.3 Multidisciplinary Input

Participants described drawing on occupational therapy and speech and language therapy input to inform complex assessments, while others cautioned against over‐reliance on multidisciplinary involvement and emphasised psychologists' core assessment competencies.

Views on multidisciplinary input were more mixed, with participants highlighting both potential benefits and limitations. For example: “Again, I think it will depend what we are assessing as to the need for input from other professionals. There are also pro's and con's of psychology being ‘gatekeepers’.” (P05).

Taken together, these findings suggest that assessment challenges are influenced not only by methodological limitations but also by wider structural constraints within services.

### 3.3 Delphi Consensus Results

These thematic findings directly guided the development of Delphi statements, with areas of strongest consensus (Table 2) highlighting the need for cross‐cultural assessment tools, enhanced approaches to adaptive functioning, and clearer guidance for interpreter‐mediated assessment.

**TABLE 2:** Statements that reached consensus agreement.

Domain	Question number	Final consensus rating	Statement	Round included
Priorities for research	2.1	89.5%	Validation of existing cognitive assessments with different cultures.	2
	2.2	100%	Development of cognitive assessments for which culture specific normative data is not required.	3
	2.3	89.4%	Validation of cognitive assessments that can be used with an interpreter.	2
	2.4	100%	Research on the impact of trauma on cognitive functioning.	2
Research into adaptive functioning	3.1	90%	Validation of adaptive behaviour questionnaires cross culturally.	2
	3.2	88.2%	Research on the reliability and validity of adaptive behaviour assessments through the use of an interpreter.	3
	3.3	89.4%	Further research in cultural differences in the domain of ‘Conceptual skills’.	2
	3.4	100%	Further research in cultural differences in the domain of ‘Social skills’.	2
	3.5	89.5%	Further research into cultural differences in the domain of ‘Practical skills’.	2
Policy and guidelines	6.1	94.1%	Assessment criteria and policy should move away from needing a definite measure of IQ and should move towards formulation‐based understanding of difficulties to reflect the complexity of assessing this client group.	3
Clinical practice and clinical context	8.1	89.5%	Assessments from other professionals (e.g., occupational therapy and speech and language therapy) should be utilised in this population to inform assessment.	2
	10.1	100%	Due to the complexity of these assessments, additional clinical time needs to be allocated to them.	2
	10.2	89.4%	The same interpreter should be booked for assessment sessions.	2
	10.3	94.8%	Extra time needs to be spent instructing the interpreter on how the assessment works and the importance of translating word‐for‐word answers.	2
	10.5	94.8%	Psychologists should receive specific training in conducting assessments through interpreters.	2
	10.6	94.8%	Psychologists should receive more training in cultural differences in understanding of intellectual disabilities.	2

*Note:* Green indicates statements that reached consensus agreement.

The results of the Delphi rounds are presented in Table 2, with 16 of the 32 statements (50%) achieving expert consensus. The items were grouped into four categories: Priorities for Research, Research into Adaptive Functioning, Policy and Guidelines, and Clinical Practice. A total of 7 statements failed to reach consensus in Round 2 and were therefore presented again in Round 3. Three of these statements then reached consensus, while the remaining statements did not. Table 3 details the statements that did not reach agreement.

**TABLE 3:** Statements that did not reach consensus agreement.

Domain	Question number	Final consensus rating	Statement	Round excluded
Priorities for research	4.1	82.3%	More research looking at the experiences of this population of being assessed in the UK.	3
	4.2	68.4%	More research looking at the experiences of family members of this population being assessed in the UK.	2
Policy and guidelines	5.1	70%	Policy and guidelines need to contain more detail on what it means to use clinical judgement in the assessment of this population.	3
Clinical practice and clinical context	8.2	82.3%	Cultural brokers (i.e., interpreters, family members, etc.) should be spoken to gather more information on specific cultural differences of the client.	3
	8.3	68.4%	It should be assessed in clinical interview how the person has adapted to life in the UK as part of assessing adaptive functioning (if relevant for the individual's background).	2
	9.1	68.4%	Cognitive assessments can be used in non‐standardised ways to gain qualitative information for assessment (i.e., through an interpreter).	2
	9.2	68.5%	It is appropriate to give a provisional diagnosis or non‐diagnosis in this population and agree to retest in a few years' time if appropriate.	2
	9.3	68.4%	Diagnosis of intellectual disability can be made without a definitive IQ score if evidence suggests the person is failing to cope with the intellectual demands of their environment.	2
	9.4	63.1%	Assessments with this client group require more flexibility with cut‐off scores and quantitative information.	2
	10.4	68.4%	Interpreters should receive basic training in interpreting during clinical assessments.	2
	10.7	82.3%	More psychologists with ethnically diverse backgrounds should be employed within intellectual disability services.	3
	11.1	26.4%	It is difficult to do these assessments due to lack of clinical time available in the current clinical context in which I work or have worked.	2
	11.2	36.9%	It is difficult to find extra time to instruct an interpreter before a session in the current clinical context in which I work or have worked.	2
	11.3	42.1%	The costs to services of purchasing additional assessments that are normed for cross‐cultural use is a barrier to assessing this population.	2
	11.4	53.1%	Assistant psychologists should not be assessing intellectual disability in this population.	2
	11.5	21.1%	Trainee psychologists should not be assessing intellectual disability in this population.	2

*Note:* Red indicates statements that did not reach consensus of agreement.

## 4 Discussion

To our knowledge, this is the first Delphi study to systematically examine UK psychologists' views on assessing intellectual disability in adults from minoritised ethnic and linguistic backgrounds. Across rounds, experts reached consensus that the field would benefit from prioritising: (i) the development and validation of cross‐culturally applicable (“culture‐free”) cognitive tools, (ii) empirical evaluation of interpreter‐mediated cognitive assessment, and (iii) substantially improved service‐level conditions to support high‐quality work (e.g., increased time allocation, interpreter continuity, and clinician training). These priorities align with wider cross‐cultural neuropsychology recommendations that emphasise assessment approaches that explicitly account for language, education, and migration history, and a move away from assuming that tests normed on Western, educated populations will generalise fairly across groups (Franzen et al. 2022; Fernández and Abe 2018; O'Donald and Calia 2025b).

A consistent theme across the qualitative material was that current practice in this area is characterised by ambiguity and inconsistency, with clinicians expressing uncertainty about what constitutes defensible practice when standardised tools cannot be used in standard ways. This reflects longstanding concerns that cross‐cultural assessment is vulnerable to cultural bias and construct‐irrelevant variance (Ardila 2005; Poortinga 1995), and that intellectual disability, as operationalised in many high‐income contexts, may rest on culturally situated assumptions about functioning and adaptation (Whitaker 2013). This ambiguity also aligns with our recent findings that clinicians often lack clear guidance on what constitutes defensible practice in cross‐cultural assessments, particularly when standardised tools cannot be applied as intended (Calia, Della Sala, et al. 2026). Importantly, clinicians emphasised that diagnostic uncertainty is not solely a technical problem: it is shaped by structural constraints, including limited time, inconsistent access to trained interpreters, and a lack of appropriate assessment tools, all of which risk amplifying existing inequalities in access to assessment and services (Robertson et al. 2019). This reliance on clinical judgement may be understood not just as a pragmatic response, but as highlighting the limitations of current measurement models when used across diverse cultural settings.

The panel strongly endorsed the need for improved training in cultural competence and interpreter‐mediated assessment, while also noting that training alone is insufficient if services cannot provide the conditions required to apply this knowledge in practice. This distinction aligns with work emphasising that health inequities are often structurally produced and cannot be addressed solely through individual‐level competence (Smith et al. 2025). In our study, consensus on additional clinical time, interpreter continuity, and targeted training suggests that cultural competence should be understood as a service‐level capability rather than an individual attribute.

The clearest research priority identified was strengthening the cross‐cultural and psychometric foundations of intellectual disability assessment. However, the notion of “culture‐free” assessment warrants critical consideration. Cognitive testing is inherently situated within cultural, educational, and linguistic contexts, and attempts to remove such influences might risk obscuring rather than resolving underlying sources of bias. In our study, experts supported both the validation of existing cognitive measures across cultural groups and the development of assessment approaches that reduce reliance on culturally embedded knowledge. This aligns with broader positions in cross‐cultural neuropsychology, which hold that progress lies in developing genuinely cross‐culturally applicable approaches rather than relying on race‐ or ethnicity‐based norms (Franzen et al. 2022). The findings also reinforce the distinction between “non‐verbal” and “culture‐free”: even tests with reduced language demands may remain culturally patterned by educational experience, test familiarity, and expectations regarding speed and accuracy (Shuttleworth‐Edwards 2016). This is particularly relevant in the UK, where non‐verbal alternatives are recommended when the WAIS‐IV cannot be validly administered, despite evidence that such tools may not be inherently culture‐free (BPS 2015).

Relatedly, experts prioritised research into the validity and reliability of interpreter‐mediated cognitive assessment. Clinically, services often face a constrained choice between testing through an interpreter, using alternative tools of uncertain validity, or declining formal testing and relying on qualitative formulation. The cross‐cultural neuropsychology literature indicates that interpreter‐mediated assessment may introduce methodological challenges that cannot be resolved through ad hoc adaptations alone (Calia, Nielsen, et al. 2026; Fujii et al. 2022). Clearer empirical guidance on when interpreter‐mediated testing is defensible and how associated risks can be mitigated would address a significant gap in current practice.

Adaptive functioning emerged as a second major research priority. Experts emphasised that adaptive behaviour is inherently culturally embedded and that existing measures may inadvertently pathologise culturally patterned roles, expectations, and opportunities. This aligns with theoretical work showing that adaptive functioning must be interpreted relative to culturally appropriate expectations rather than universal norms (Calia et al. 2020; Tassé et al. 2012). Concerns about the limitations of questionnaire‐based measures echo critiques that many scales capture culturally shaped skills rather than the underlying construct of adaptive behaviour (Balboni et al. 2022). Given ongoing international efforts to adapt and validate adaptive behaviour measures across contexts, UK‐based research could usefully focus on how these tools perform when used with interpreters and across diverse educational and migration histories.

In contrast, research focused on the experiences of individuals and families undergoing assessment received less consistent endorsement, despite being raised in qualitative responses. This mirrors broader trends in the field, where professional‐led agendas have historically dominated and lived‐experience research is only beginning to gain traction (Jacobs et al. 2023). Consensus on the impact of trauma on cognitive functioning further underscores clinicians' concerns about differential diagnostic uncertainty in contexts where trauma exposure and post‐migration adversity are plausible confounds, aligning with broader cross‐cultural literature cautioning against interpreting low cognitive scores as stable indicators of intellectual impairment without careful consideration of contextual factors (Nielsen et al. 2024).

At a policy level, findings point to the need for clearer, more operational guidance. Clinicians reported substantial variation in how cognitive testing is conducted when English language proficiency is limited, raising the risk that access to diagnosis becomes contingent on broader systemic factors rather than need. Although existing guidance acknowledges the role of clinical judgement, mixed responses to statements about specifying its use reflect an unresolved tension between standardisation for equity and the irreducible complexity of these assessments. This echoes Whitaker's critique of rigid thresholds and assumptions about measurement precision in intellectual disability diagnosis (Whitaker 2008), and tensions between standardisation and person‐centred judgement have been reported in UK interpreter‐mediated paediatric neuropsychological assessment (O'Donald and Calia 2025b).

Service‐level resourcing was also central to many consensus statements. Additional time, interpreter continuity, and protected space for preparation and formulation were considered prerequisites for defensible practice. These findings reinforce the argument that equitable assessment requires structural adaptation, not merely improved individual competence (O'Hara 2003). Multidisciplinary working divided opinion, but suggestions that occupational therapy and speech and language therapy input may enhance understanding of functional abilities are consistent with evidence that observation‐based approaches can provide valuable complementary information during complex assessments (Mahoney et al. 2021), provided such input is integrated within a coherent psychological formulation.

Several limitations should be considered when interpreting this study's findings. First, some non‐consensus statements may have been too broad or context‐dependent to be uniformly endorsed; future Delphi work might benefit from allowing participants to refine or reword items between rounds to improve specificity. Additionally, the relatively small and professionally homogenous sample may limit the breadth of perspectives captured, particularly from services with differing levels of exposure to culturally and linguistically diverse populations. The composition of the expert panel, which was predominantly drawn from clinical psychology, may also have shaped the perspectives represented, potentially limiting the inclusion of alternative professional or regional viewpoints. Second, the panel was weighted towards NHS Scotland; expertise from regions with higher linguistic diversity may have produced different priorities or stronger consensus on certain operational issues. This may reflect service‐specific practices and resource constraints, which could influence the priorities identified. Third, in Round 3, only items that had not reached consensus were re‐rated, which is consistent with common Delphi practice but limits analysis of response stability across rounds. As such, the stability of responses across all items could not be fully examined, which may limit conclusions about the consistency of agreement. Additionally, using both positively and negatively worded items may reduce acquiescence bias but can introduce method effects and impact reliability; therefore, consensus rates for negatively phrased items should be interpreted cautiously. The study also relied on self‐reported practice, which may be influenced by recall bias or social desirability. In addition, the study did not capture service user or family perspectives, limiting conclusions about how assessment practices are experienced by those undergoing the process. This is especially important given the cultural and contextual aspects of assessment, and future research should focus on including lived experience to ensure findings represent both clinical and service user perspectives. Finally, different cut‐offs may have produced different patterns of agreement, so non‐consensus items should not be interpreted as unimportant, but rather as reflecting areas of genuine uncertainty or contested practice within the field.

## 5 Conclusions

This study offers a structured account of how experienced clinicians conceptualise and prioritise the challenges involved in assessing intellectual disability in culturally and linguistically diverse adults within the UK context. Experts converged on the need for (i) more robust cross‐cultural cognitive and adaptive assessment options, (ii) empirical clarification of interpreter‐mediated assessment, (iii) clearer guidance to support equitable practice, and (iv) service resourcing to make high‐quality assessment feasible. Collectively, the findings highlight that addressing inequity requires both methodological advances and structural change. At a theoretical level, the study reinforces a core dilemma: intellectual disability is widely understood as a human condition, yet the tools used to operationalise diagnosis are culturally situated and largely normed on Western, educated, English‐speaking populations. Experts' calls for less culturally embedded tools and more context‐sensitive assessment approaches reflect both an ethical imperative to reduce marginalisation and a scientific need to minimise construct‐irrelevant variance in diagnosis.

## Funding

The authors have nothing to report.

## Ethics Statement

Ethical approval for this study was obtained from the University of Edinburgh, School of Health in Social Science Ethics Committee. Generic research and development approval was also obtained from NHS Lothian.

## Consent

Informed consent was obtained from all participants prior to participation. All participants received a participant information sheet and provided consent before completing the study surveys.

## Conflicts of Interest

The authors declare no conflicts of interest.

## Data Availability

The datasets generated and analysed during the current study are not publicly available due to the risk of participant identification, but are available from the corresponding author on reasonable request.

## References

[jar70279-bib-0001] Allison, L. , and A. Strydom . 2009. “Intellectual Disability Across Cultures.” Psychiatry 8, no. 9: 355–357.

[jar70279-bib-0002] AlMuhairy, O. , E. Efthymiou , H. ElHoweris , M. Alshathly , and A. Sartawi . 2023. “Development of the Emirati Child Adaptation Scale (ECAS) for Assessing the Behavioral Adaptation Skills of Children With and Without Disabilities in the UAE.” Education in Science 13, no. 4: 406.

[jar70279-bib-0004] Ardila, A. 2005. “Cultural Values Underlying Psychometric Cognitive Testing.” Neuropsychology Review 15, no. 4: 185–195. 16395623 10.1007/s11065-005-9180-y

[jar70279-bib-0005] Baker, J. , K. Lovell , and N. Harris . 2006. “How Expert Are the Experts? An Exploration of the Concept of “Expert” Within Delphi Panel Techniques.” Nurse Researcher 14, no. 1: 59–70. 10.7748/nr2006.07.14.1.59.c6025. 17100214

[jar70279-bib-0006] Balboni, G. , A. Bacherini , P. Anselmi , et al. 2022. “Italian Diagnostic Adaptive Behavior Scale: Reliability and Diagnostic Accuracy Compared With the Vineland‐II.” Research in Developmental Disabilities 123: 104185. 10.1016/j.ridd.2022.104185. 35190325

[jar70279-bib-0007] Beiderbeck, D. , N. Frevel , and H. A. von der Gracht . 2021. “Preparing, Conducting, and Analyzing Delphi Surveys: Cross‐Disciplinary Practices, New Directions, and Advancements.” MethodsX 8: 101401. 10.1016/j.mex.2021.101401. 34430297 PMC8374446

[jar70279-bib-0008] Birukou, A. , E. Blanzieri , P. Giorgini , F. Giunchiglia , F. Ricci , and M. Fumagalli . 2013. “A Formal Definition of Culture for the Semantic Web.” Journal of Web Semantics 19: 1–21.

[jar70279-bib-0009] British Psychological Society . 2015. Guidance on the Assessment and Diagnosis of Intellectual Disabilities in Adulthood. BPS. www.bps.org.uk/guideline/guidance‐assessment‐and‐diagnosis‐intellectual‐disabilities‐adulthood.

[jar70279-bib-0010] British Psychological Society . 2017. Guidelines for Psychologists Working With Interpreters. BPS. www.bps.org.uk/guideline/working‐interpreters‐guidelines‐psychologists.

[jar70279-bib-0059] Calia, C. , S. Della Sala , F. O’Donald , M. Forrest , and M. Brazzelli . 2026. “DECODE: An Innovative Tool to Aid Cross‐Linguistic Neuropsychological Assessment.” Clinical Neuropsychologist: 1–17. 10.1080/13854046.2026.2651220. 41934197

[jar70279-bib-0011] Calia, C. , T. R. Nielsen , S. Franzen , T. Watermeyer , and N. Mukadam . 2026. “Interpreter‐Mediated Neuropsychological Assessment.” In Working With Interpreters in Mental Health, edited by A. Editor and B. Editor , 2nd ed., 16. Routledge.

[jar70279-bib-0012] Calia, C. , F. O'Donald , S. Franzen , et al. 2025. “Cross‐Cultural Functional Assessment for Dementia: A Commentary.” Clinical Neuropsychologist: 1–26. 10.1080/13854046.2025.2543913. 40819654

[jar70279-bib-0013] Calia, C. , E. Pickett , F. O'Donald , and M. A. Parra . 2020. “Functional Assessment of Cognitively Impaired Older Adults: Are We Asking the Right Questions?” Alzheimer's & Dementia 16: e044948.

[jar70279-bib-0014] Durà‐Vilà, G. , and M. Hodes . 2009. “Ethnic Variation in Service Utilisation Among Children With Intellectual Disability.” Journal of Intellectual Disability Research 53, no. 11: 939–948. 19732279 10.1111/j.1365-2788.2009.01214.x

[jar70279-bib-0015] Earley, N. 2015. A Consensus Approach Towards Identifying Pertinent Therapist Characteristics in Good Lives Model Treatment: A Research Portfolio. Thesis, University of Edinburgh. http://hdl.handle.net/1842/21705.

[jar70279-bib-0016] Emerson, E. 2012. “Deprivation, Ethnicity and the Prevalence of Intellectual and Developmental Disabilities.” Journal of Epidemiology & Community Health 66, no. 3: 218–224. 20889590 10.1136/jech.2010.111773

[jar70279-bib-0017] Emerson, E. , S. Azmi , C. Hatton , A. Caine , R. Parrott , and J. Wolstenholme . 1997. “Is There an Increased Prevalence of Severe Learning Disabilities Among British Asians?” Ethnicity & Health 2, no. 4: 317–321. 9526694 10.1080/13557858.1997.9961840

[jar70279-bib-0018] Fernández, A. L. , and J. Abe . 2018. “Bias in Cross‐Cultural Neuropsychological Testing: Problems and Possible Solutions.” Culture and Brain 6: 1–35.

[jar70279-bib-0019] Förster, B. , and H. A. von der Gracht . 2014. “Assessing Delphi Results in Strategic Foresight: A Comparison of Panels and Polled Experts.” Technological Forecasting and Social Change 87: 24–39. 10.1016/j.techfore.2013.07.016.

[jar70279-bib-0020] Franzen, S. , European Consortium on Cross‐Cultural Neuropsychology (ECCroN) , T. J. Watermeyer , et al. 2022. “Cross‐Cultural Neuropsychological Assessment in Europe: Position Statement of the European Consortium on Cross‐Cultural Neuropsychology (ECCroN).” Clinical Neuropsychologist 36, no. 3: 546–557. 34612169 10.1080/13854046.2021.1981456

[jar70279-bib-0021] Fujii, D. , O. Santos , and L. Della Malva . 2022. “Interpreter‐Assisted Neuropsychological Assessment: Clinical Considerations.” In Understanding Cross‐Cultural Neuropsychology, 135–147. Routledge.

[jar70279-bib-0022] Jacobs, P. , K. Watchman , H. Wilkinson , L. Hoyle , and L. McGenily . 2023. “Experiences of People With Intellectual Disability and Dementia: A Systematic Review.” Journal of Applied Research in Intellectual Disabilities 36, no. 2: 241–258. 36562340 10.1111/jar.13063 PMC10107172

[jar70279-bib-0023] Kapadia, D. , and H. Bradby . 2021. “Ethnicity and Health.” In Routledge International Handbook of Critical Issues in Health and Illness, 183–196. Routledge.

[jar70279-bib-0024] Kapadia, D. , J. Zhang , S. Salway , et al. 2022. “Ethnic Inequalities in Healthcare: A Rapid Evidence Review.” www.nhsrho.org/wp‐content/uploads/2023/05/RHO‐Rapid‐Review‐Final‐Report_pdf.

[jar70279-bib-0025] Keeney, S. , F. Hasson , and H. McKenna . 2010. The Delphi Technique in Nursing and Health Research. Wiley‐Blackwell.

[jar70279-bib-0026] King, N. 2012. “Doing Template Analysis.” In Qualitative Organizational Research: Core Methods and Current Challenges, edited by G. Symon and C. Cassell , 426–450. SAGE.

[jar70279-bib-0027] Lam, J. , R. Aldridge , R. Blackburn , and K. Harron . 2023. “How Is Ethnicity Reported, Described, and Analysed in Health Research in the UK? A Bibliographical Review and Focus Group Discussions With Young Refugees.” BMC Public Health 23, no. 1: 2025. 10.1186/s12889-023-16948-1. 37848866 PMC10583485

[jar70279-bib-0028] Leonard, H. , and X. Wen . 2002. “The Epidemiology of Intellectual Disability: Challenges and Opportunities.” Mental Retardation and Developmental Disabilities Research Reviews 8, no. 3: 117–134. 12216056 10.1002/mrdd.10031

[jar70279-bib-0029] Liebkind, K. 2006. “Ethnic Identity and Acculturation.” In The Cambridge Handbook of Acculturation Psychology, 78–96. Cambridge University Press.

[jar70279-bib-0030] Mahoney, W. J. , M. G. Blaskowitz , and K. R. Johnson . 2021. “Occupational Therapy–Related Assessments for Adults With Intellectual Disability: A Scoping Review.” American Journal of Occupational Therapy 75, no. 3: 7503180100. 10.5014/ajot.2021.046342. 34781342

[jar70279-bib-0031] Maulik, P. K. , M. N. Mascarenhas , C. D. Mathers , T. Dua , and S. Saxena . 2011. “Prevalence of Intellectual Disability: A Meta‐Analysis.” Research in Developmental Disabilities 32, no. 2: 419–436. 21236634 10.1016/j.ridd.2010.12.018

[jar70279-bib-0032] McGrother, C. W. , S. Bhaumik , C. F. Thorp , J. M. Watson , and N. A. Taub . 2002. “Prevalence and Service Need Among South Asian and White Adults With Intellectual Disability.” Journal of Intellectual Disability Research 46, no. 4: 299–309. 12000581 10.1046/j.1365-2788.2002.00391.x

[jar70279-bib-0058] Metcalfe, T. , T. R. Nielsen , F. O’Donald , S. Franzen , and C. Calia . 2026. “A Systematic Review of the Measurement and Influence of Quality of Education on Neuropsychological Test Performance in Later Life.” Neuropsychology Review: 1–22. 10.1007/s11065-025-09689-z. 41697598

[jar70279-bib-0033] Mindt, M. R. , A. Arentoft , K. Coulehan , et al. 2019. “Neuropsychological Evaluation of Culturally/Linguistically Diverse Older Adults.” In Handbook on the Neuropsychology of Aging and Dementia, 25–48. Springer International Publishing.

[jar70279-bib-0034] Nasa, P. , R. Jain , and D. Juneja . 2021. “Delphi Methodology in Healthcare Research: How to Decide Its Appropriateness.” World Journal of Methodology 11, no. 4: 116–129. 10.5662/wjm.v11.i4.116. 34322364 PMC8299905

[jar70279-bib-0035] NHS Race and Health Observatory . 2023. “We Deserve Better: Ethnic Minorities With a Learning Disability and Access to Healthcare.” https://nhsrho.org/research/review‐into‐factors‐that‐contribute‐towards‐inequalities‐in‐health‐outcomes‐faced‐by‐those‐with‐a‐learning‐disability‐from‐a‐minority‐ethnic‐community/.

[jar70279-bib-0036] Nielsen, T. R. , S. Franzen , T. Watermeyer , et al. 2024. “Interpreter‐Mediated Neuropsychological Assessment: Clinical Considerations and Recommendations From the European Consortium on Cross‐Cultural Neuropsychology (ECCroN).” Clinical Neuropsychologist 38, no. 8: 1775–1805. 38588670 10.1080/13854046.2024.2335113

[jar70279-bib-0037] Nielsen, T. R. , and M. Staios . 2023. “Cognitive Assessment in Recently Arrived Migrants: Limitations of Non‐Verbal Tests.” Clinical Neuropsychologist 37, no. 2: 1–15.34791971

[jar70279-bib-0038] Oakland, T. , D. Iliescu , H. Y. Chen , and J. H. Chen . 2013. “Cross‐National Assessment of Adaptive Behavior.” Journal of Psychoeducational Assessment 31, no. 5: 435–447.

[jar70279-bib-0039] O'Donald, F. , and C. Calia . 2025a. “Preliminary Psychometric Evaluation of the Vancouver Index of Acculturation (VIA) in a UK‐Based East‐Asian Sample.” Scandinavian Journal of Psychology 67: 257–265. 40984032 10.1111/sjop.70029

[jar70279-bib-0040] O'Donald, F. , and C. Calia . 2025b. “Interpreter‐Mediated Paediatric Neuropsychological Assessments: Clinician and Interpreter Experiences and Consensus‐Based Recommendations.” Journal of Neuropsychology 20: 231–245. 41284193 10.1111/jnp.70024

[jar70279-bib-0041] O'Donald, F. , C. Calia , and S. Franzen . 2025. “Cross‐Cultural Paediatric Neuropsychological Assessment: Key Considerations.” Health Science Reports 8, no. 6: e70959. 40551860 10.1002/hsr2.70959 PMC12183384

[jar70279-bib-0042] Office for National Statistics . 2021. “Census 2021: Ethnic Group, England and Wales.” www.ons.gov.uk/peoplepopulationandcommunity/culturalidentity/ethnicity/bulletins/ethnicgroupenglandandwales/census2021.

[jar70279-bib-0043] O'Hara, J. 2003. “Learning Disabilities and Ethnicity: Achieving Cultural Competence.” Advances in Psychiatric Treatment 9, no. 3: 166–174.

[jar70279-bib-0044] Poortinga, Y. H. 1995. “Cultural Bias in Assessment: Historical and Thematic Issues.” European Journal of Psychological Assessment 11, no. 3: 140–146.

[jar70279-bib-0045] Roberts, K. , A. Dowell , and J. B. Nie . 2019. “Attempting Rigour and Replicability in Thematic Analysis of Qualitative Research Data; a Case Study of Codebook Development.” BMC Medical Research Methodology 19, no. 1: 1–8. 30922220 10.1186/s12874-019-0707-y PMC6437927

[jar70279-bib-0046] Robertson, J. , R. Raghavan , E. Emerson , S. Baines , and C. Hatton . 2019. “Health and Healthcare of People With Intellectual Disabilities From Minority Ethnic Groups in the UK: A Systematic Review.” Journal of Applied Research in Intellectual Disabilities 32, no. 6: 1310–1334. 31169955 10.1111/jar.12630

[jar70279-bib-0047] Rowe, G. , and G. Wright . 1999. “The Delphi Technique as a Forecasting Tool: Issues and Analysis.” International Journal of Forecasting 15, no. 4: 353–375. 10.1016/S0169-2070(99)00018-7.

[jar70279-bib-0048] Schalock, R. L. 2004. “Adaptive Behaviour: Its Conceptualisation and Measurement.” In The International Handbook of Applied Research in Intellectual Disabilities, edited by E. Emerson , C. Hatton , T. Thompson , and T. R. Parmenter , 369–384. 10.1002/9780470713198.ch18.

[jar70279-bib-0049] Shuttleworth‐Edwards, A. 2016. “Generally Representative Is Representative of None.” Clinical Neuropsychologist 30, no. 7: 975–998. 27377008 10.1080/13854046.2016.1204011

[jar70279-bib-0050] Smith, M. , F. O´ Donald , L. J. Sevier‐Guy , and A. Heffernan . 2025. “The Barriers and Enablers to Implementing Trauma‐Informed Care.” British Dental Journal 239, no. 1: 21–24. 40646205 10.1038/s41415-025-8906-x

[jar70279-bib-0051] Sonderlund, A. L. , F. Baygi , J. Soendergaard , and T. Thilsing . 2024. “Advancing Health Equity for Populations With Intellectual Disabilities: A Systematic Review of Facilitators and Barriers to the Implementation of Health Checks and Screening.” SSM ‐ Health Systems 2: 100009.

[jar70279-bib-0052] Stancliffe, R. J. , and K. C. Lakin . 2006. “Longitudinal Trends in Health Service Use by People With Intellectual Disabilities.” Journal of Intellectual Disability Research 50, no. 6: 469–478.

[jar70279-bib-0053] Tassé, M. J. , R. L. Schalock , G. Balboni , et al. 2012. “The Construct of Adaptive Behavior: Its Conceptualization, Measurement, and Use in the Field of Intellectual Disability.” American Journal on Intellectual and Developmental Disabilities 117, no. 4: 291–303. 22809075 10.1352/1944-7558-117.4.291

[jar70279-bib-0054] Whitaker, S. 2008. “Intellectual Disability: A Concept in Need of Revision?” British Journal of Developmental Disabilities 54, no. 106: 3–9.

[jar70279-bib-0055] Whitaker, S. 2013. Intellectual Disability: An Inability to Cope With an Intellectually Demanding World. Springer.

[jar70279-bib-0056] Whitaker, S. 2018. “The Future of Services for People With Intellectual Disabilities.” Tizard Learning Disability Review 23, no. 2: 85–92.

